# Triggered ferroptotic albumin-tocopherol nanocarriers for treating drug-resistant breast cancer

**DOI:** 10.3389/fonc.2024.1464909

**Published:** 2024-10-23

**Authors:** Qianqian Gao, Tingting Liu, Li Sun, Yongliang Yao, Fang Li, Lingxiang Mao

**Affiliations:** ^1^ Department of Laboratory Medicine, Affiliated Kunshan Hospital of Jiangsu University, Kunshan, Jiangsu, China; ^2^ Science and Technology Talents, Affiliated Kunshan Hospital of Jiangsu University, Kunshan, Jiangsu, China

**Keywords:** ferroptosis, photodynamic therapy, biocompatibility, drug-resistant breast cancer, albumin nanocarrier, indocyanine green

## Abstract

Ferroptosis is considered an effective method to overcome drug-resistant tumors. This study aims to use three FDA-approved biological materials, human serum albumin, D-α-tocopherol succinate, and indocyanine green, to construct a novel biocompatible nanomaterial named HTI-NPs, exploring its effect in drug-resistant breast cancer (MCF-7/ADR cells). The research results indicate that HTI-NPs can selectively inhibit the proliferation of MCF-7/ADR cells *in vitro*, accompanied by upregulating transferrin receptor, generating reactive oxygen species, and downregulating glutathione peroxidase 4. Under laser irradiation, HTI-NPs can promote ferroptosis by inhibiting glutathione expression through photodynamic therapy. Notably, HTI-NPs exhibit good inhibitory effects on MCF-7/ADR xenograft tumors *in vivo*. In conclusion, HTI-NPs represent a biocompatible nanomaterial that induces ferroptosis, providing new insights and options for treating drug-resistant breast cancer.

## 1 Introduction

Breast cancer is the most common and deadliest malignancy among women. Since 2020, there have been 2.3 million diagnosed female breast cancer patients globally, with 685,800 deaths attributed to breast cancer. It is projected that by 2040, the total number of breast cancer patients worldwide will increase by 50% (1, 2). With the advancement of various treatment modalities such as radiation therapy, chemotherapy, surgical resection, and targeted therapy, the overall mortality rate of breast cancer has decreased by approximately 30% to 40%, and the 5-year survival rate for breast cancer patients after early intervention is as high as 90% (3, 4). However, inherent and acquired drug resistance remains a challenging issue in triple-negative breast cancer and advanced breast cancer. It is also a primary cause of chemotherapy failure and mortality in breast cancer patients. Finding effective measures to overcome drug-resistant breast cancer is crucial for further improving patient survival rates.

Ferroptosis, unlike traditional apoptosis, is considered a form of iron-dependent, lipid peroxidation-driven, and programmed cell death (5). In ferroptosis, excessive reactive oxygen species (ROS) not only induce lethal lipid peroxidation but also interact with biomolecules such as DNA/RNAs and proteins, causing irreversible negative effects on the cell. Based on these characteristics, ferroptosis is considered an effective method to overcome drug-resistant tumors (6–8).

In this study, a novel ferroptosis-inducing nanomaterial called HTI-NPs was designed using human serum albumin (HSA), D-α-tocopherol succinate (TOS), and indocyanine green (ICG). TOS is one of the most potent anticancer derivatives of vitamin E. It rapidly induces a large amount of ROS by acting on mitochondrial respiratory chain complex II and disrupting the electron transport chain, thereby exerting cytotoxic effects on various tumor cells, including breast cancer, while being relatively nontoxic to normal cells (9–12). Baratz et al. found that vitamin E can regulate the intracellular iron ion content, thus affecting the redox state within cells (13). Therefore, it is speculated that TOS has the potential to induce ferroptosis in tumor cells.

Furthermore, TOS-based nanomaterials possess characteristics such as self-assembly, ease of manipulation, particle size suitable for intravenous injection, and passive targeting mediated by the enhanced permeability and retention (EPR) effect, making them widely used in the field of anticancer nanomaterial research (14). HSA, the most abundant endogenous protein in serum, has advantages such as good biocompatibility, low cost, long half-life, and high yield. It is extensively used in the preparation of nanocarriers. The hydrophobic structure of HSA can bind with drug molecules (such as paclitaxel) and other materials (such as TOS), making it suitable for constructing multifunctional nanomaterials (15–18).

ICG is an FDA-approved near-infrared fluorescent dye used in clinical practice for liver function testing and medical imaging (19, 20). Studies have found that ICG can also exert photodynamic therapy (PDT) effects under specific near-infrared light wavelengths (approximately 800 nm) by producing lethal ROS to kill tumor cells (21, 22). However, direct use of ICG is limited by its unstable aqueous solution, concentration-dependent self-aggregation, short half-life (2-4 minutes), and lack of targeting ability (23, 24). To overcome these limitations, various ICG-based nanomaterials have been designed and prepared to enhance stability, targeting capability, and prolong the half-life, thereby maximizing its anticancer activity (25–27). Furthermore, ICG can be adsorbed and bound to HSA through hydrophobic interactions (28). In summary, based on the interactions between HSA, TOS, and ICG, as well as the self-assembly properties of TOS, we synthesized nanoparticles (HTI-NPs) with TOS and HSA as the framework and ICG attached to the surface. We hypothesize that these nanoparticles can enhance ferroptosis by inducing ROS in tumor cell mitochondria through TOS, and mediating PDT through ICG, thereby exerting an anti-drug-resistant breast cancer effect.

**Figure 1:**
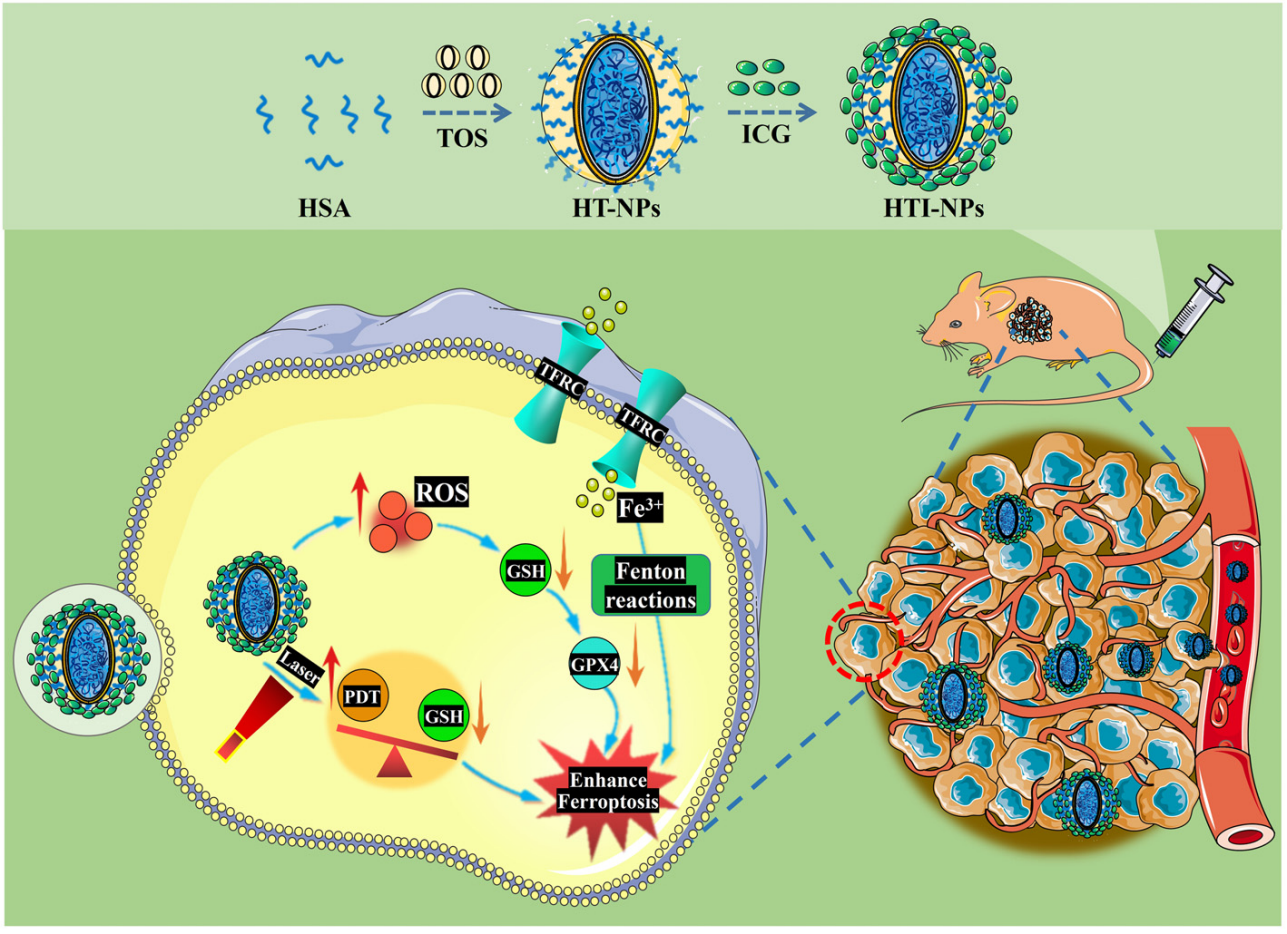
Schematic illustration of the preparation of HTI-NPs by self-assembly between HSA, TOS, and ICG; Combined PDT-ferroptosis therapy *in vitro* and vivo. HSA, human serum albumin; TOS, D-α-tocopherol Succinate; ICG, indocyanine green; HT-NPs, HSA-TOS nanoparticles; HTI-NPs, HSA-TOS-ICG nanoparticles; PDT, photodynamic therapy; ROS, reactive oxygen species; TFRC, transferrin receptor; GPX4, glutathione peroxidase 4; GSH, glutathione.

As shown in **Figure 1**, following the established methods in our research group, HTI-NPs were synthesized, and their cytotoxic effects on drug-resistant breast cancer and the associated mechanisms of ferroptosis were investigated. The results showed that HTI-NPs could target tumor tissues and selectively kill drug-resistant breast cancer cells. The potential mechanism of action may involve induction of ROS, upregulation of transferrin receptor (TFRC), and downregulation of glutathione peroxidase 4 (GPX4), leading to ferroptosis. This study has developed a novel ferroptosis-inducing nanomaterial, providing an ideal platform for the treatment of drug-resistant breast cancer.

## 2 Materials and methods

### 2.1 Materials

Albumin human (HSA) was obtained from Sigma-Aldrich (St Louis, MO, USA). N-hydroxysuccinimide (NHS), 1-(3-dimethylaminopropyl)-3-ethylcarbodiimide (EDC) and D-α-tocopherol Succinate (TOS) were purchased from Aladdin Chemistry Co., Ltd (Shanghai, China). Indocyanine green (ICG) was supplied from Tokyo Chemical Industry Co., Ltd (Japan). Bromcresol Green (BCG) assay was purchased from BioRike (Changsha, China). MCF-10A special medium was supplied from Procell (Wuhan, China). DMEM, LIVE/DEAD TM Cell Imaging Ki, DAPI, fetal bovine serum (FBS), trypsin and penicillin-streptomycin were all obtained from Thermo Fisher Scientific (Waltham, MA, USA). The Cell Counting Kit-8 (CCK-8) assay was supplied from Dojindo (Kumamoto, Japan). Rsduced Glutathione (GSH) Assay Kit was purchased from Solarbio (Beijing, China). EdU Cell Proliferation Kit was obtained from CellorLabTM (Shanghai, China). Bicinchoninic acid (BCA) protein assay kit and ROS assay kit (DCFH-DA) were obtained from the Beyotime Institute of Biotechnology (Shanghai, China). For Western blotting analysis, GAPDH antibody were obtained from Abcam (Cambridge, MA, USA), while those for GPX4 and TFRC antibody were purchased from ABclonal (Wuhan, China). All other reagents, which were reagent grade or better, were supplied from commercial sources and used as received.

### 2.2 Synthesis and characterization of HTI-NPs

Preparation of HTI-NPs: 5 mg of HSA was dissolved in 10 mL of deionized water. 30 mg of TOS was dissolved in 1 mL of ethanol, and 10 mg of ICG was dissolved in 1 mL of DMSO. EDC (21.67 mg) and NHS (13.01 mg) were added to the TOS solution, and the mixture was reacted for 10 minutes. The 10 mL HSA aqueous solution was placed in a 50 mL centrifuge tube, and under stirring conditions, 100 µL of the TOS (EDC/NHS) solution was added dropwise to the HSA solution. After stirring for 30 minutes, 50 µL of the ICG solution was added dropwise to the aforementioned mixture. The solution was stirred in the dark for 210 minutes and then centrifuged for 20 minutes at 14,800 rpm to collect the nanoparticles. The collected nanoparticles were washed three times with deionized water by centrifugation, and the obtained HSA-TOS -ICG nanoparticles (abbreviated as HTI-NPs) were dispersed in 10 mL of deionized water for further use.

Characterization of HTI-NPs: The nanoparticle suspension was drop-casted onto carbon-coated copper grids for observation under a transmission electron microscope (TEM; H-7800, HTIachi, Japan). The average diameter and zeta potential of the nano-particles were measured using Malvern Zetasizer Nano ZS90 (Malvern Instruments, Malvern, UK). The absorbance spectrum of the nanoparticles was observed using a UV-visible spectrophotometer (T6 New Century, Beijing Pgeneral, China). To quantitatively measure the ICG content in HTI-NPs, 1 mL of the nanoparticle suspension was centrifuged at 14,800 rpm for 20 minutes to remove the supernatant. The pellet was then dissolved in DMSO to completely disrupt the nanoparticles and release the loaded ICG. The absorbance of ICG at 780 nm was measured using an enzyme-linked immunosorbent assay (ELISA) reader (Bio-Rad, USA). The original data were converted to ICG concentration using a reference standard curve (data not shown). The concentration of HSA in HTI-NPs was determined using the bromocresol green (BCG) reagent kit. The concentration of TOS was calculated as the total weight of freeze-dried nanoparticles minus the weight of ICG and HSA.

### 2.3 Cell culture and animals

Human breast cancer cells (MCF-7 cells) were sourced from Chinese Academy of Sciences Cell Bank (Shanghai, China). Human multidrug-resistant breast cancer cells (MCF-7/ADR cells) were obtained from Cancer Institute of the Second Affiliated Hospital of Zhejiang University School of Medicine (Zhejiang, China). Human normal liver cells (HL-7702 cells) and human normal mammary epithelial cells (MCF-10A cells) were sourced from BNCC (Kunshan, China). MCF-7, MCF-7/ADR, and HL-7702 cells were cultured in DMEM medium containing 10% (v/v) fetal bovine serum (FBS) and 1% (v/v) antibiotics (penicillin-streptomycin, 100 U/mL) at 37°C with 5% CO2. MCF-10A cells were cultured in MCF-10A specific medium.

Four-week-old female BALB/c nude mice were obtained from the Laboratory Animal Technology Co., Ltd. of Hangzhou Ziyuan (Zhejiang, China) and housed under specific pathogen-free (SPF) conditions. All the animal procedures complied with the guidelines of the Laboratory Animal Ethics Committee of Jiangsu University and were approved by the Jiangsu University ethics committee.

### 2.4 Cellular uptake of HTI-NPs

Firstly, we observed the distribution of HTI-NPs inside cells using a confocal laser scanning microscope. MCF-7/ADR cells (1×10^5^ cells/well) were seeded onto 6-well plates with 20×20 mm glass coverslips. After cell attachment, the cells were treated with fresh culture medium containing free ICG or HTI-NPs (ICG concentration of 5 µg/mL) and incubated for 4 hours. Subsequently, the cells were carefully washed three times with PBS and fixed with 4% (w/v) paraformaldehyde for 15 minutes. The para-formaldehyde was then removed, and the cells were washed three times with PBS. The cell nuclei were stained with DAPI for 5 minutes, followed by three washes with PBS. The coverslips were mounted for observation. Finally, the cellular uptake and intracellular localization of ICG were observed using a confocal laser scanning microscope (GE Healthcare, Issaquah, WA, USA).

In addition, we quantitatively analyzed the cellular uptake of HTI-NPs using flow cytometry. MCF-7/ADR cells (1×10^5^ cells/well) were seeded onto 6-well plates. After cell attachment, the cells were treated with free ICG and HTI-NPs for 4 hours, followed by three washes with PBS. The cells were then dissociated with trypsin and centrifuged. The cells were resuspended in PBS and analyzed using a flow cytometer (FACScan, Becton Dickinson, USA). The fluorescence signal of ICG was analyzed in the FL-4 channel. The acquired data were analyzed using FlowJo_V10 software.

The cell uptake procedure for MCF-7 cells was the same as for MCF-7/ADR cells, as described above.

### 2.5 Cell viability test

Firstly, the *in vitro* therapeutic effect of HTI-NPs was examined. In brief, MCF-10A cells, MCF-7 cells, MCF-7/ADR cells, and HL-7702 cells (5×10^3^ cells per well) were seeded into 96-well plates and incubated at 37°C with 5% CO_2_ for 24 hours until the cells were fully adhered. Then, the cells were exposed to HTI-NPs in culture medium at different concentrations. After 24 hours of incubation, cell viability was determined using the CCK-8 assay.

Next, the combined therapeutic effect of HTI-NPs and photodynamic therapy (PDT) was evaluated. In brief, MCF-7 cells and MCF-7/ADR cells were exposed to free ICG and HTI-NPs (ICG concentration of 5 µg/mL) for 4 hours, followed by irradiation with an 808 nm laser (2 W/cm^2^, 5 min) or no irradiation. Subsequently, after 24 hours of cell culture, cell viability was measured using the CCK-8 assay.

### 2.6 Intracellular ROS detection by HTI-NPs

To evaluate the intracellular ROS levels induced by HTI-NPs, MCF-10A cells, MCF-7 cells, MCF-7/ADR cells, and HL-7702 cells (2×10^4^ cells per well) were evenly seeded in 24-well plates and incubated overnight at 37°C with 5% CO_2_ until the cells were fully adhered. Then, the cells were further cultured with fresh medium containing HTI-NPs (ICG concentration of 5 µg/mL). After 24 hours of incubation, the cells were washed to remove residual HTI-NPs and treated with DCFH-DA probe according to the instructions. DCFH-DA is non-fluorescent until it is oxidized by intracellular ROS to form DCF (dichlorofluorescein), which emits green fluorescence. The fluorescence signal inside the cells was observed using a fluorescence microscope to detect the intracellular ROS levels induced by HTI-NPs.

To measure the intracellular ROS concentration after laser treatment (808 nm, 2 W/cm^2^, continuous irradiation for 5 minutes), MCF-7 cells and MCF-7/ADR cells were exposed to free ICG and HTI-NPs (ICG concentration of 5 µg/mL) for 4 hours, followed by either receiving or not receiving 808 nm laser irradiation. Subsequently, the ROS levels were measured as described above. The mean fluorescence intensity of ROS was quantitatively analyzed using Image J software.

### 2.7 Mechanism of ferroptosis induced by HTI-NPs

For GSH determination, MCF-7/ADR cells were cultured in 6-well plates (1×10^6^ cells per well) and incubated with complete medium, complete medium + laser, ICG, ICG + laser, HTI-NPs, and HTI-NPs + laser (808 nm, 2 W/cm^2^, continuous irradiation for 5 minutes, ICG concentration of 5 µg/mL) for 24 hours. Afterward, the total GSH level was measured using a GSH assay kit.

For Western blotting studies, MCF-7/ADR cells (5×10^5^ cells per well) were placed in 6-well plates and incubated overnight at 37°C with 5% CO_2_. The cells were exposed to HTI-NPs at different concentrations for 24 hours, washed three times with cold PBS, and lysed with RIPA lysis buffer containing 1% phenylmethylsulfonyl fluoride. The lysates were incubated on ice for 15 minutes and then centrifuged at 4°C for 15 minutes to collect the proteins. The protein concentration was determined using a BCA protein assay kit. The extracted proteins were analyzed by SDS-PAGE and transferred onto PVDF membranes. The membranes were incubated overnight at 4°C with specific primary antibodies (against GPX4, TFRC, or GAPDH) and then incubated with secondary antibodies. Finally, the immunoreactive bands were visualized using the MiniChemi Mini Size Chemiluminescent Imaging System (Beijing Sage Creation Science Co., Ltd) and quantified by Image J software (National Institutes of Health, Bethesda, MD, USA).

After laser treatment (808 nm, 2 W/cm^2^) for 5 minutes, the levels of GPX4 or TFRC were detected. In summary, MCF-7 cells and MCF-7/ADR cells were co-incubated with free ICG and HTI-NPs (ICG concentration of 5 µg/mL) for 4 hours, followed by irradiation with or without an 808 nm laser (2 W/cm^2^, continuous irradiation for 5 minutes). Subsequently, GPX4 or TFRC levels were measured using the methods described above.

### 2.8 EdU detection

The inhibitory effect of HTI-NPs on the proliferation of MCF-7/ADR cells was evaluated using the EdU Cell Proliferation Assay Kit with Alexa Fluor 555. Briefly, cells (2×10^4^ cells per well) were seeded in different treatment wells of a 24-well plate. After 24 hours of incubation, the corresponding concentration of EdU solution was added to each well and incubated for 3 hours. Subsequently, the cells were fixed with 4% paraformaldehyde for 20 minutes and permeabilized with PBS containing 0.3% Triton X-100 for 20 minutes. The reaction solution was then added in the dark and incubated for 30 minutes. Finally, the cells were stained with DAPI to visualize the cell nuclei. Cell images were captured using a fluorescence microscope (Carl Zeiss, Germany). The nuclei of proliferating cells were stained with EdU and appeared as red fluorescence. The cell proliferation was quantitatively analyzed using Image J software.

### 2.9 Live/Dead staining

The combined therapeutic effect of HTI-NPs and photodynamic therapy (PDT) was further evaluated using a live/dead cell staining assay. In brief, MCF-7/ADR cells (2×10^4^ cells per well) were seeded in a 24-well plate and incubated overnight. The cells were then co-incubated with complete medium, complete medium + laser, ICG, ICG + laser, HTI-NPs, and HTI-NPs + laser (808 nm, 2 W/cm^2^, continuous irradiation for 5 minutes, ICG concentration of 5 µg/mL) for 24 hours. Afterward, the cells were stained using the LIVE/DEAD Cell Imaging Kit. The stained cells were observed under a fluorescence microscope (Carl Zeiss, Germany) to visualize the live cells (green) and dead cells (red). The number of live and dead cells was quantitatively analyzed using Image J software.

### 2.10 *In vivo* biodistribution via fluorescence imaging

First, an MCF-7/ADR tumor xenograft model was established by subcutaneously injecting 2 × 10^7^ MCF-7/ADR cells into the right flank of 4-6-week-old female BALB/c nude mice. When the tumor volume reached 60-100 mm^3^, the mice were randomly divided into two groups (n=3): the ICG group and the HTI-NPs group. Subsequently, ICG and HTI-NPs (ICG concentration of 100 µg/mL, volume of 200 µL) were intravenously injected into the mice through the tail vein. At 2, 8, and 24 hours after injection, *in vivo* fluorescence imaging was performed using an *in vivo* imaging system (IVIS, Burker *in-vivo* Xtreme II) (excitation wavelength: 750 nm, emission wavelength: 830 nm). After 24 hours of injection, the mice were euthanized, and their major organs, including the heart, liver, spleen, lungs, kidneys, and tumors, were collected and imaged using IVIS.

### 2.11 *In vivo* antitumor effect of the HTI-NPs

When the tumor volume reached 60-100 mm3, the mice were randomly divided into four groups (n≥5). The treatment groups included PBS, ICG + laser, HTI-NPs, and HTI-NPs + laser (808 nm, 2 W/cm^2^, continuous irradiation for 5 minutes, equivalent to an ICG concentration of 100 µg/mL and a volume of 200 µL per mouse). The mice were treated every two days for a total of four administrations, and the tumor volume and body weight changes were recorded over a period of 10 days. The tumor volume was measured using the following formula: V = L × W^^2^ × 0.5, where L represents the longest diameter of the tumor and W represents the shortest diameter of the tumor. At 24 hours after administration, the tumor area was irradiated with 808 nm laser (power density of 2 W/cm^2^, lasting for 5 minutes). At the end of the treatment, all mice were euthanized, and the tumor tissues were collected. Therapeutic efficacy was assessed by H&E staining and immunohistochemical staining (TFRC and GPX4). Additionally, major organs including the heart, liver, spleen, lungs, and kidneys were collected and subjected to H&E staining to evaluate the toxicity of HTI-NPs on normal tissues.

### 2.12 Statistical analysis

The results are presented as mean ± standard deviation (SD) using GraphPad Prism 9.5. Group comparisons were assessed using the unpaired Student’s t-test. One-way analysis of variance (ANOVA) was used to compare the differences between more than two groups. Differences between groups were considered statistically significant at a level of P < 0.05 (*).

## 3 Results

### 3.1 Synthesis and characterization of HTI-NPs

**Figure 2:**
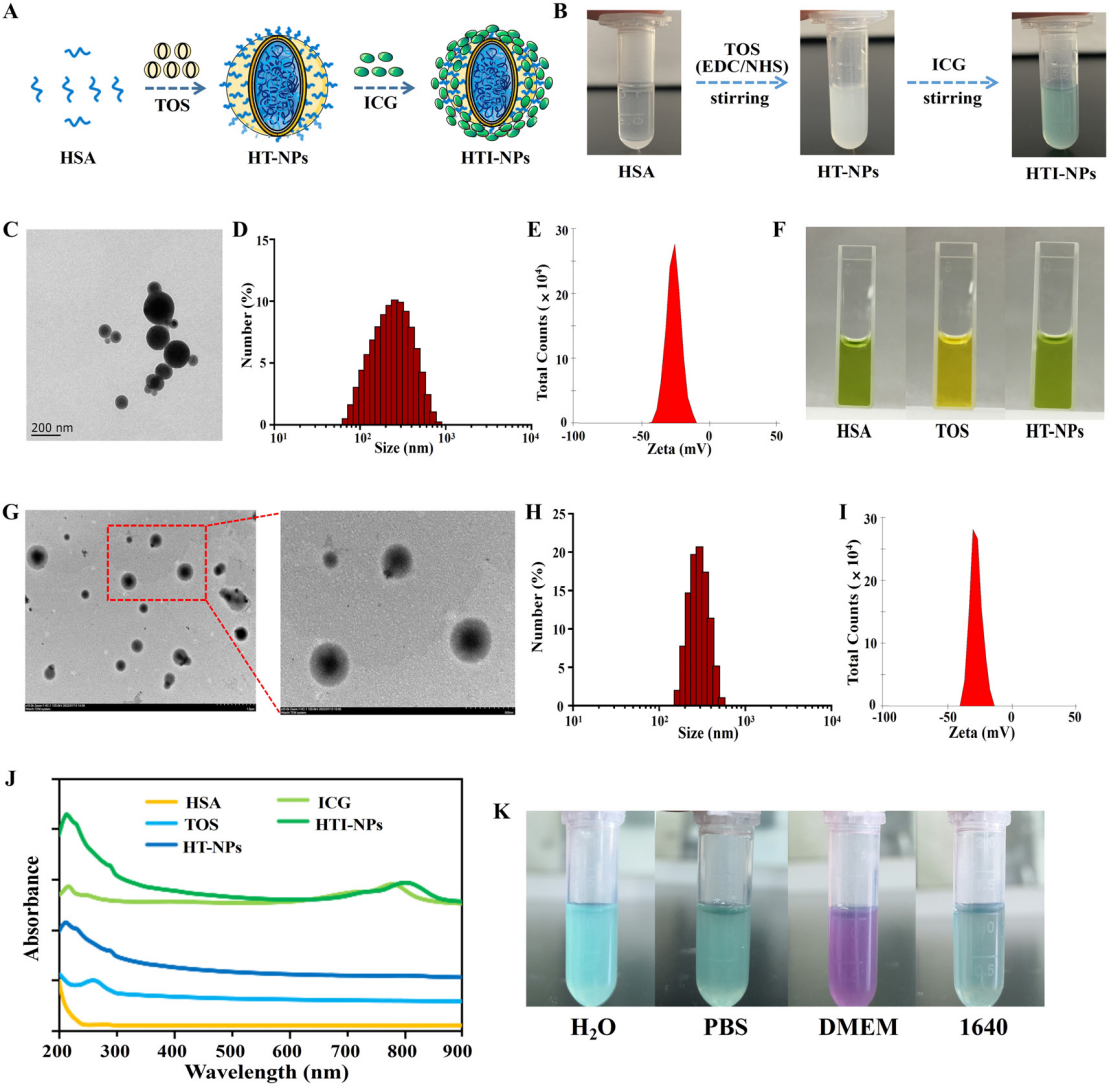
**(A)** Schematic illustration of the preparation of HTI-NPs. **(B)** Photographs of the preparation of HTI-NPs. **(C)** TEM images, **(D)** Particle size and **(E)** zeta potential of HT-NPs. **(F)** HAS, TOS, and HAS-TOS NPs with BCG reagent. **(G)** TEM images, **(H)** Particle size and **(I)** zeta potential of HTI-NPs. **(J)** UV-vis absorption spectra of HAS, TOS, ICG, HT-NPs, HTI-NPs. **(K)** HTI-NPs distributed into H_2_O solution, PBS solution, DMEM medium, and 1640 medium. BCG, bromocresol green.

**Figure 2A** provided a description of the HTI-NPs preparation procedure. As shown in **Figure 2B**, the colorless, translucent fluid turned to an opaque, milky dispersion after the addition of TOS, showing that TOS was used to create the nanoparticles. The UV-vis-NIR spectra result had an absorption peak near 300 nm that was almost identical to that of TOS (**Figure 2J**), indicating that TOS was present in them. **Figure 2F** demonstrates that whereas the TOS group appeared negative yellow, the HSA and HT-NPs groups appeared positive green, indicating the presence of HSA in the nanoparticles. TEM imaging showed that HT-NPs were spherical with good monodispersity (**Figure 2C**). The HT-NPs mean diameter was found to be 210 ± 4.0 nm and the zeta potential was -26.9 ± 1.2 mV (**Figures 2D, E**).

On this basis, after centrifugation/washing, the solution changed from its original opaque milky dispersion to green solution (**Figure 2B**), confirming the incorporation of ICG in nanoparticles. HTI-NPs looked spherical and monodispersed like HT-NPs, with an average diameter of 275 ± 7 nm (**Figures 2G, H**). The zeta potential was found to be -27.0 ± 1.0 mV (**Figure 2I**). As shown in **Figure 2J**, the UV-vis-NIR spectra revealed that HT-NPs showed no absorption in the NIR region. HTI-NPs displayed an absorption peak at about 780 nm, which was identified as the typical ICG absorption peak (**Figure 2J**). HTI-NPs formulation contained HSA, TOS, and ICG in amounts of 58.2%, 33.8%, and 8%, respectively. In addition, HTI-NPs were efficiently distributed into H_2_O solution, PBS solution, DMEM medium, and 1640 medium (**Figure 2K**).

### 3.2 Cellular uptake of HTI-NPs

**Figure 3:**
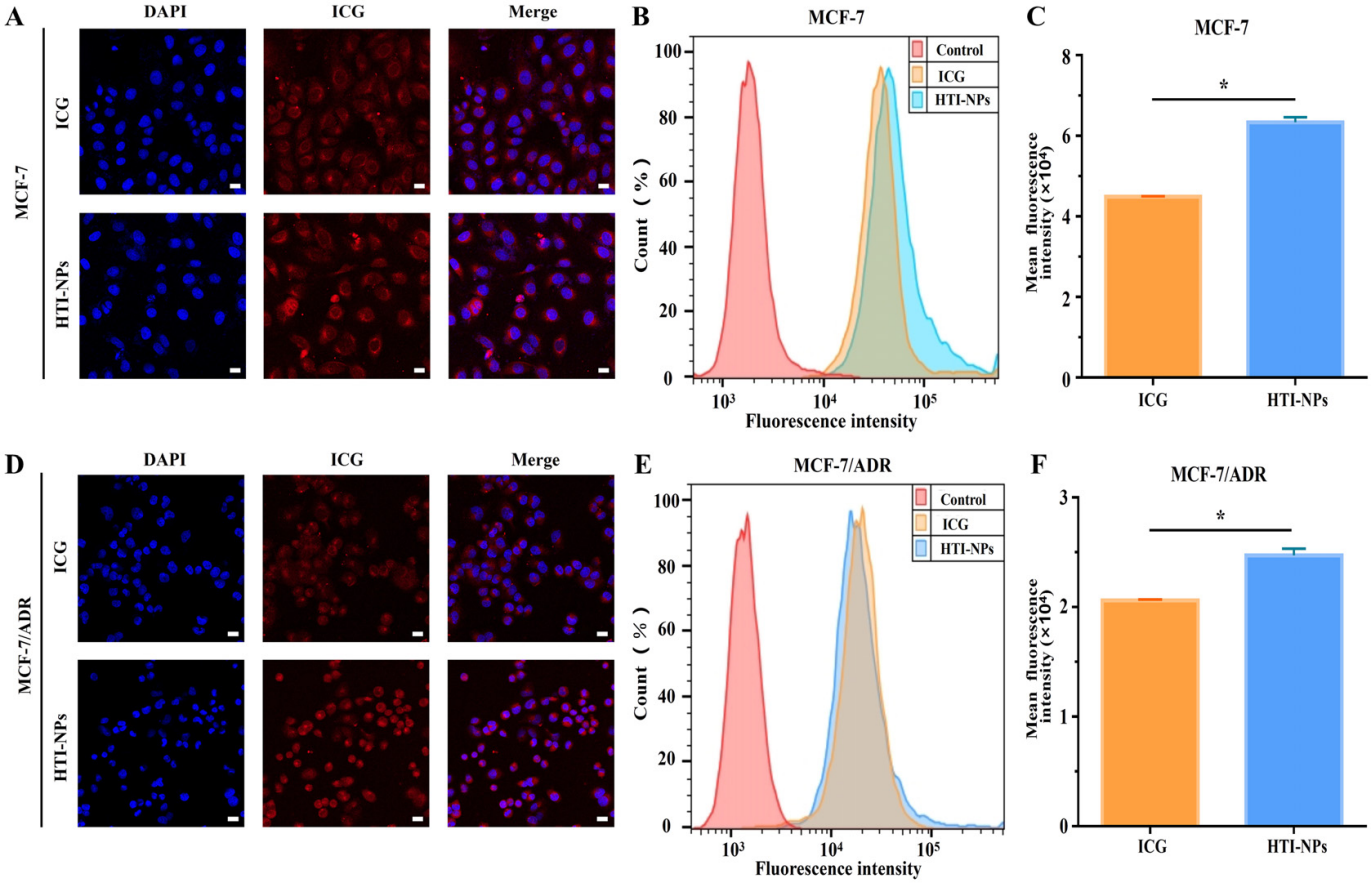
**(A)** Confocal fluorescent images, **(B)** flow cytometry analysis and **(C)** fluorescence intensity of ICG in MCF-7 cells after incubation with free ICG and HTI-NPs for 4 hours. **(D)** Confocal fluorescent images, **(E)** flow cytometry analysis and **(F)** fluorescence intensity of ICG in MCF-7/ADR cells after incubation with free ICG and HTI-NPs for 4 hours. DAPI (nuclei, blue), ICG (red). (Scale bar is 20 µm). * P < 0.05.

Firstly, the overlap of red and blue fluorescence in the free ICG group indicated that the ICG was primarily dispersed in the cytoplasm (**Figure 3A**). The **Figure 3B** data showed that MCF-7 cells had ICG fluorescent signal, proving that the cells had taken up both free ICG and HTI-NPs. The cellular uptake of HTI-NPs was obviously higher than that of free ICG due to the different cellular uptake processes of HTI-NPs and free ICG (**Figure 3C**). The same results were obtained using the human drug-resistant type MCF-7/ADR breast cancer cells as another cell model. In agreement with these observations, the fluorescence images demonstrate that ICG was detectable in the cytoplasm of MCF-7/ADR cells (**Figure 3D**). In addition, noticeably higher cellular uptake of ICG carried by HTI-NPs than that of free ICG was observed (**Figures 3E, F**).

### 3.3 *In vitro* antitumor and ferroptosis study of HTI-NPs

**Figure 4:**
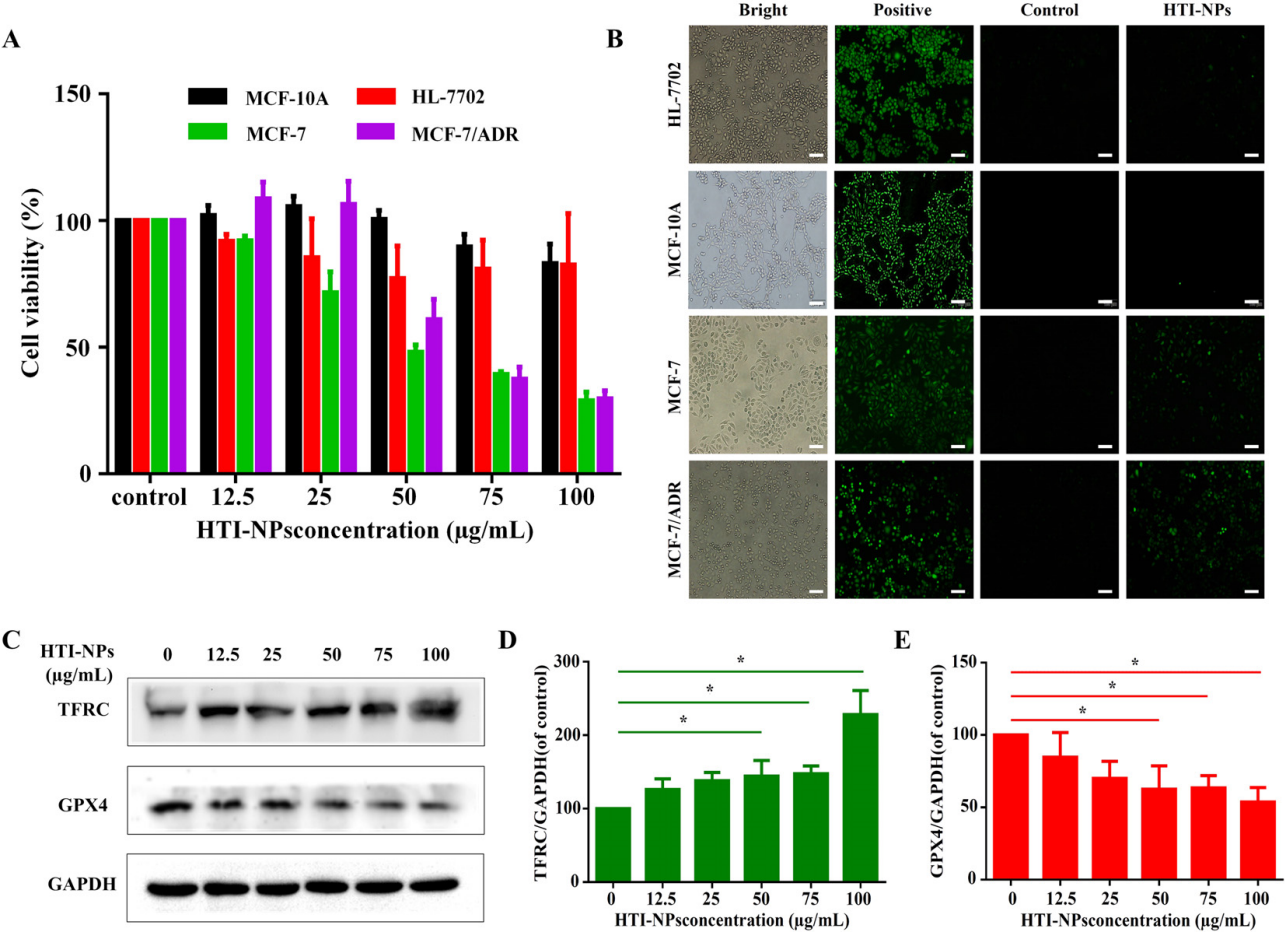
**(A)**The cytotoxicity and **(B)** ROS of HTI-NPs against MCF-10A cells, HL-7702 cells, MCF-7, and MCF-7/ADR after incubation for 24 h (Scale bar is 100 µm). **(C)** Western blotting assay was used to measure **(D)** TFRC, and **(E)** GPX4 after exposure to various concentrations of HTI-NPs. GAPDH served as a loading control. Data show mean ± standard deviation (n = 3). * P < 0.05. TFRC, transferrin receptor; GPX4, glutathione peroxidase 4.

According to **Figure 4A**, the survival rates of MCF-10A and HL-7702 cells were discovered to be not considerably lowered when cultured with HTI-NPs. More than 80% of MCF-10A and HL-7702 cells were still alive when the treatment concentration of HTI-NPs reached 100 µg/ml. Interestingly, the viability of the MCF-7 and MCF-7/ADR cells was less than 30% after treatment with 100 µg/mL HTI-NPs (**Figure 4A**).

Consistent with cellular assay results, HTI-NPs significantly increased the green fluorescence intensity of MCF-7 and MCF-7/ADR cells, whereas MCF-10A and HL-7702 cells exhibited no green fluorescence, indicating that HTI-NPs could generate ROS and cause the occurrence of ferroptosis (**Figure 4B**). We also monitored the expression level of intracellular TFRC during treatment. **Figures 4C, D** demonstrate that, in contrast to the untreated group, the expression of TFRC steadily increased as the concentration of HTI-NPs rose. This suggests that nanoparticles could activate TFRC. **Figures 4C, E** demonstrate that, in comparison to the control group, the expression level of GPX4 in tumors steadily decreased with increasing quantities of HTI-NPs.

### 3.4 *In vitro* PDT-ferroptosis combinatorial therapy of HTI-NPs

**Figure 5:**
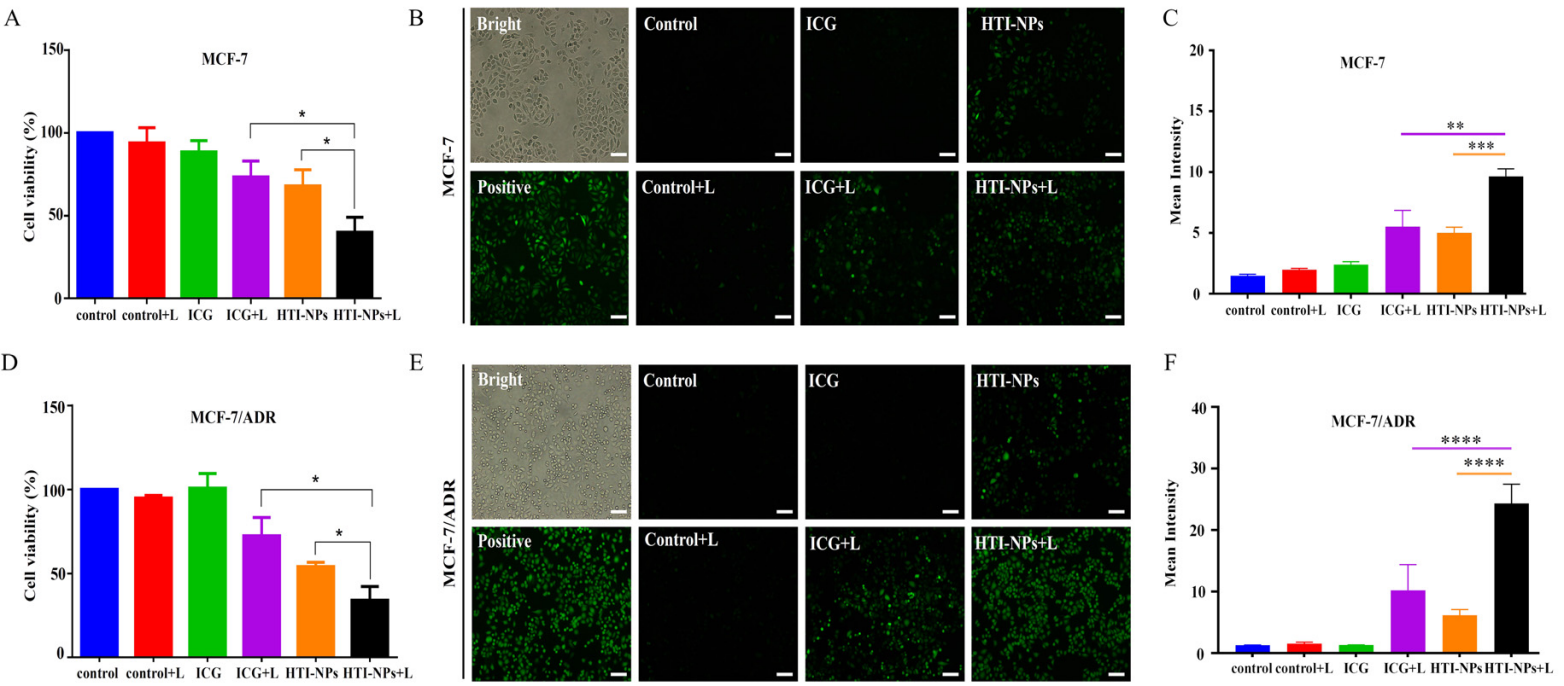
**(A)** The viability and **(B)** ROS of MCF-7 cells after treatment with free ICG and HTI-NPs without or with laser irradiation. **(C)** The quantitative analysis of **(B)**. **(D)** The viability and **(E)** ROS of MCF-7/ADR cells after treatment with free ICG and HTI-NPs without or with laser irradiation. **(F)** The quantitative analysis of **(E)**. * P < 0.05. (Scale bar is 100 µm). **P < 0.01, *** P < 0.001, ****P < 0.0001.

The survival of MCF-7 cells treated with free ICG and HTI-NPs was 88.5% and 67.9%, respectively, as shown in **Figure 5A**. After the laser irradiating, the survival rates fell to 73.2% and 40% correspondingly. **Figures 5B, C** demonstrated that under laser activation, the nanoparticles may considerably increase the formation of intracellular ROS. In contrast, green fluorescence intensity in MCF-7 cells were low after being treated by the HTI-NPs without laser irradiation or the ICG with laser irradiation, indicating that the HTI-NPs could effectively enhance ROS levels by laser stimulation. Additionally, the results of **Figure 5D** once more showed that PDT-ferroptosis combinatorial therapy was used on HTI-NPs exposed to laser radiation. Similar to each other, MCF-7/ADR cells displayed faint green signals in the cytoplasm in both the free ICG groups with laser and the HTI-NPs groups without laser (**Figures 5E, F**). The HTI-NPs groups showed the strong green signals. It demonstrated once more that HTI-NPs under laser exposure could encourage the development of ROS.

**Figure 6:**
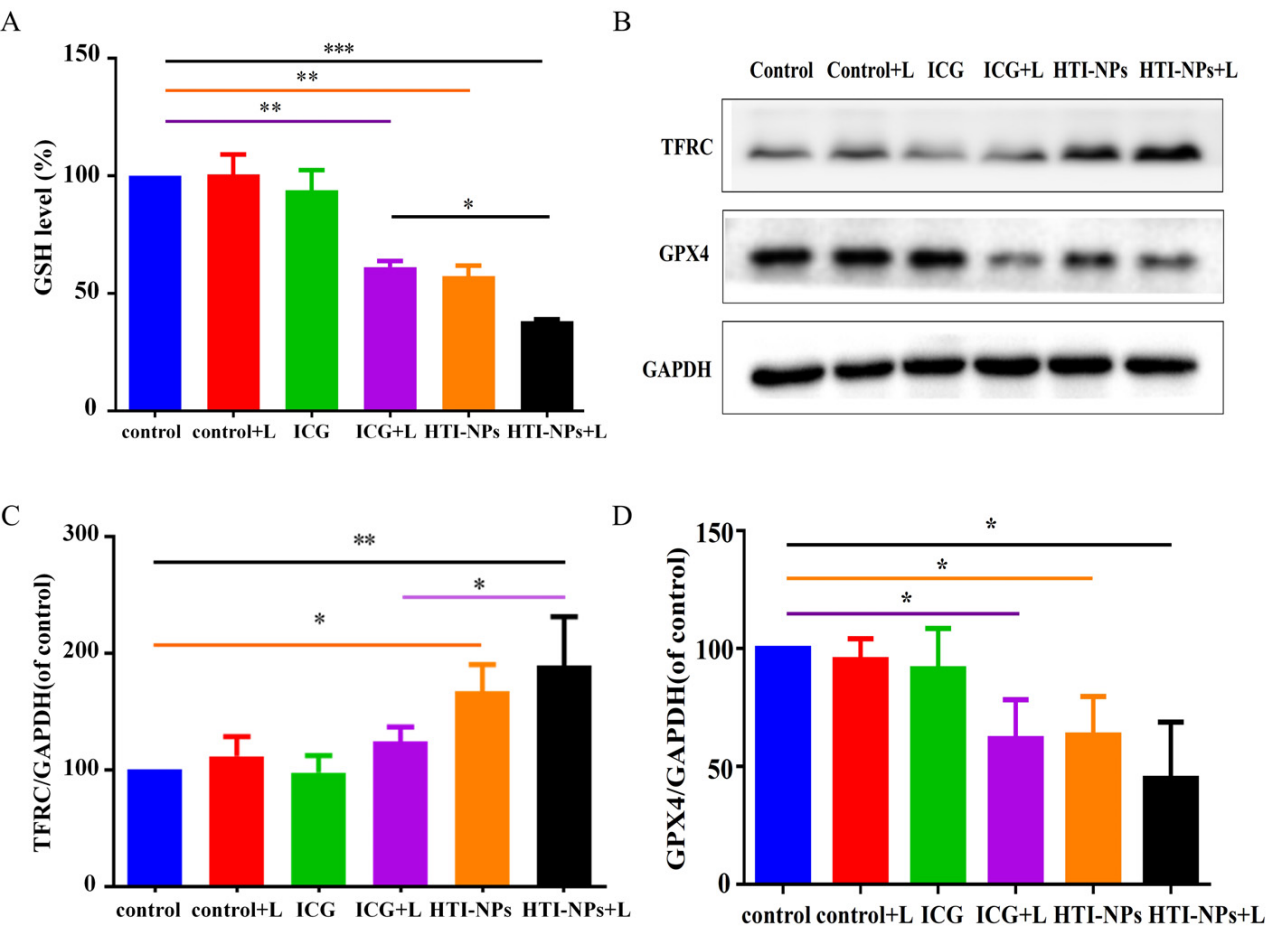
**(A)** GSH level of MCF-7/ADR cells after treatment with free ICG and HTI-NPs without or with laser irradiation. **(B)** Western blotting assay was used to measure **(C)** TFRC, and **(D)** GPX4 after exposure to free ICG and HTI-NPs without or with laser irradiation. GAPDH served as a loading control. Data show mean ± standard deviation (n = 3). * P < 0.05. GSH, glutathione. * P < 0.05, **P < 0.01,*** P < 0.001.

As seen in **Figure 6A**, compared to other treatments, the HTI-NPs with laser irradiation greatly reduced GSH in MCF-7/ADR cells. Western blotting results showed that activation of TFRC and inhibition of GPX4 activity triggered ferroptosis-associated cell death (**Figure 6B**). Therefore, we detected the expression of TFRC and GPX4 after treatment with laser irradiation. **Figures 6B, C** showed that compared with other treatment groups, HTI-NPs significantly up-regulated intracellular TFRC of MCF-7/ADR after laser irradiation. Similarly, the results of **Figures 6B, D** showed that HTI-NPs could significantly inhibit GPX4 expression under laser irradiation, further indicating that the promotion of ferroptosis by HTI-NPs was positively correlated with laser irradiation. Taken together, HTI-NPs exhibited enhanced cytotoxicity against tumor cells via PDT-ferroptosis synergistic therapy.

**Figure 7:**
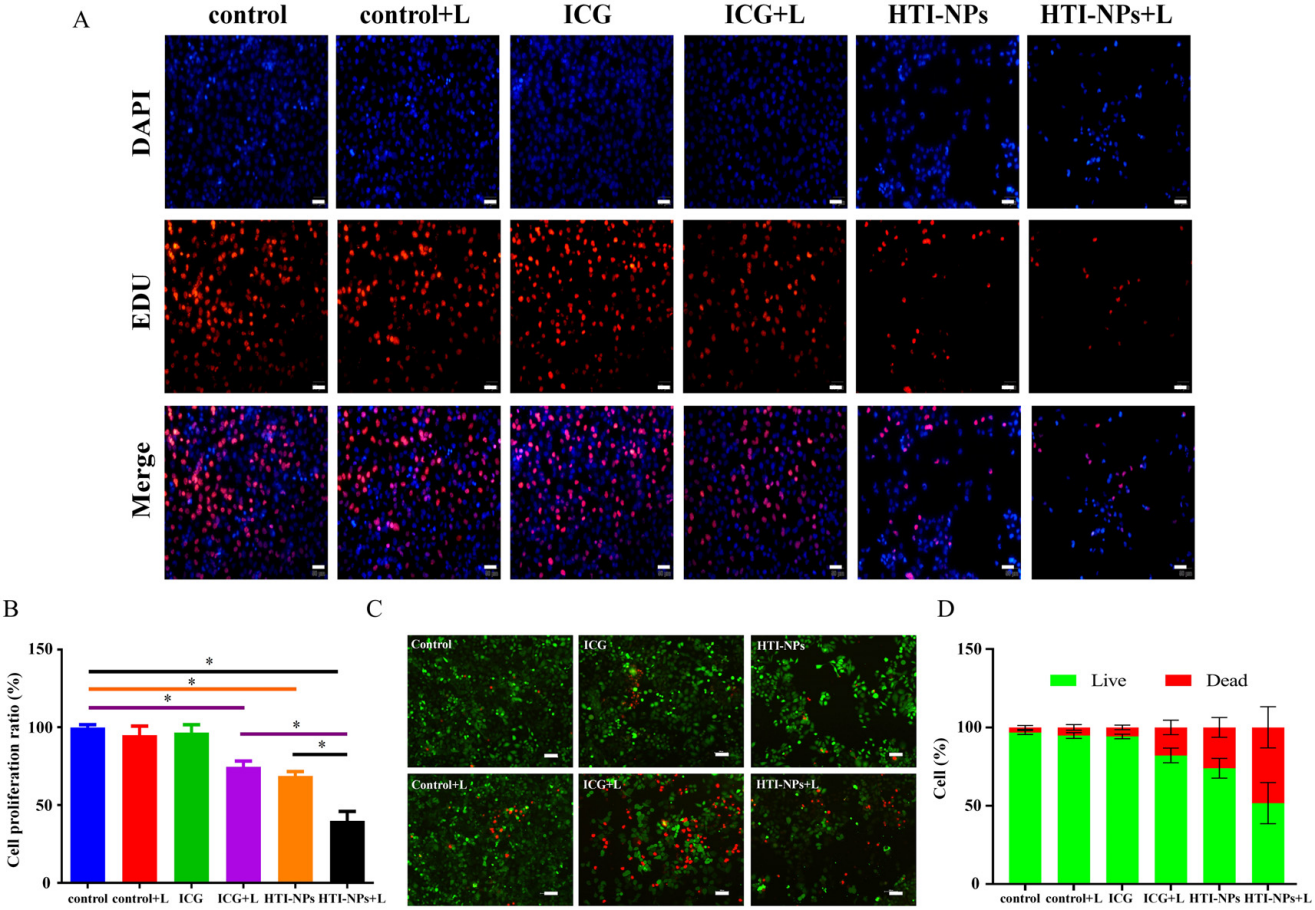
**(A, B)** MCF-7/ADR cells by Edu cell proliferation assay. (Scale bar is 50 µm). **(C)** Live/Dead staining of MCF-7/ADR cells after treatment with complete medium (control), control + Laser, free ICG, free ICG + Laser, HTI-NPs, HTI-NPs + Laser. **(D)** The quantitative analysis of **(C)**. * P < 0.05. (Scale bar is 100 µm).

As shown in **Figures 7A, B**, HTI-NPs could obviously decrease EdU-positive cell percentage under laser irradiation. The Live/Dead assay was used to subjectively assess MCF-7/ADR cells following various treatments to further demonstrate this synergistic impact. The HTI-NPs + laser group showed the most potent cell-killing impact, which was further supported by the comparison of Live/Dead assay. **Figures 7C, D** presents the findings, while the number of living cells dramatically increased in the ICG + Laser or HTI-NPs groups, just a few cells in the HTI-NPs + Laser group displayed green fluorescence, proving that the HTI-NPs had substantial cytotoxicity when exposed to laser light. These fluorescence images of Live/Dead stained assay and EdU assay intuitively illustrated the effective killing of drug-resistant cancer cells after PDT-ferroptosis therapy via HTI-NPs.

### 3.5 *In vivo* biodistribution of HTI-NPs

**Figure 8:**
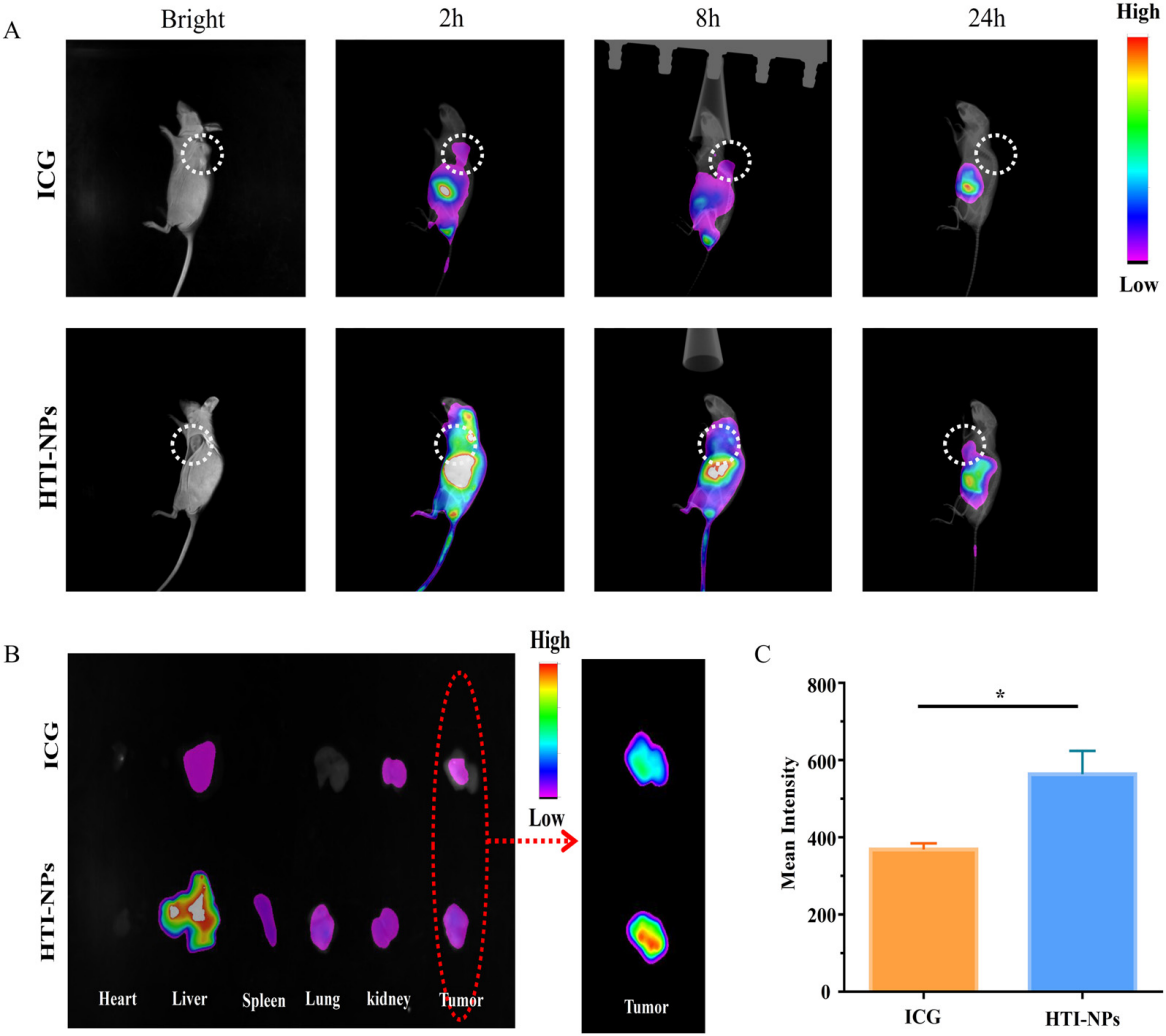
**(A)** *In vivo* fluorescence images of MCF-7/ADR tumor bearing mice at 2, 8 and 24 h after intravenous injection of ICG and HTI-NPs. **(B)** Ex vivo fluorescence images of heart, liver, spleen, lungs, kidneys, and tumors after 24 h post-injection of ICG and HTI-NPs. **(C)** Fluorescence intensity of tumor after 24 h post-injection of ICG and HTI-NPs. * P < 0.05.

**Figure 8A** displays the fluorescence signal for ICG and HTI-NPs administered systemically via intravenous injection at various time intervals. Even 24 hours after the injection, the tumor sites of HTI-NPs group still showed clear fluorescence signal, which was conducive to antitumor treatment *in vivo*. In HTI-NPs, due to the biocompatibility of HSA, nanoparticles have prolonged blood circulation time and possess nano-sized particles, which can accumulate at the tumor site through the EPR effect. The outcome was a convincing illustration of the tumor-targeting effectiveness and high retention of nanoparticles.

Meanwhile, the major organs were harvested and the ex vivo fluorescence was used to assess the distribution of the HTI-NPs. As shown in **Figures 8B, C**, the fluorescence intensity of the HTI-NPs group in the tumor was 1.53-fold higher than that of the free ICG group, indicating that the HTI-NPs may enhance tumor accumulation and *in vivo* imaging.

### 3.6 *In vivo* antitumor efficacy of HTI-NPs

**Figure 9:**
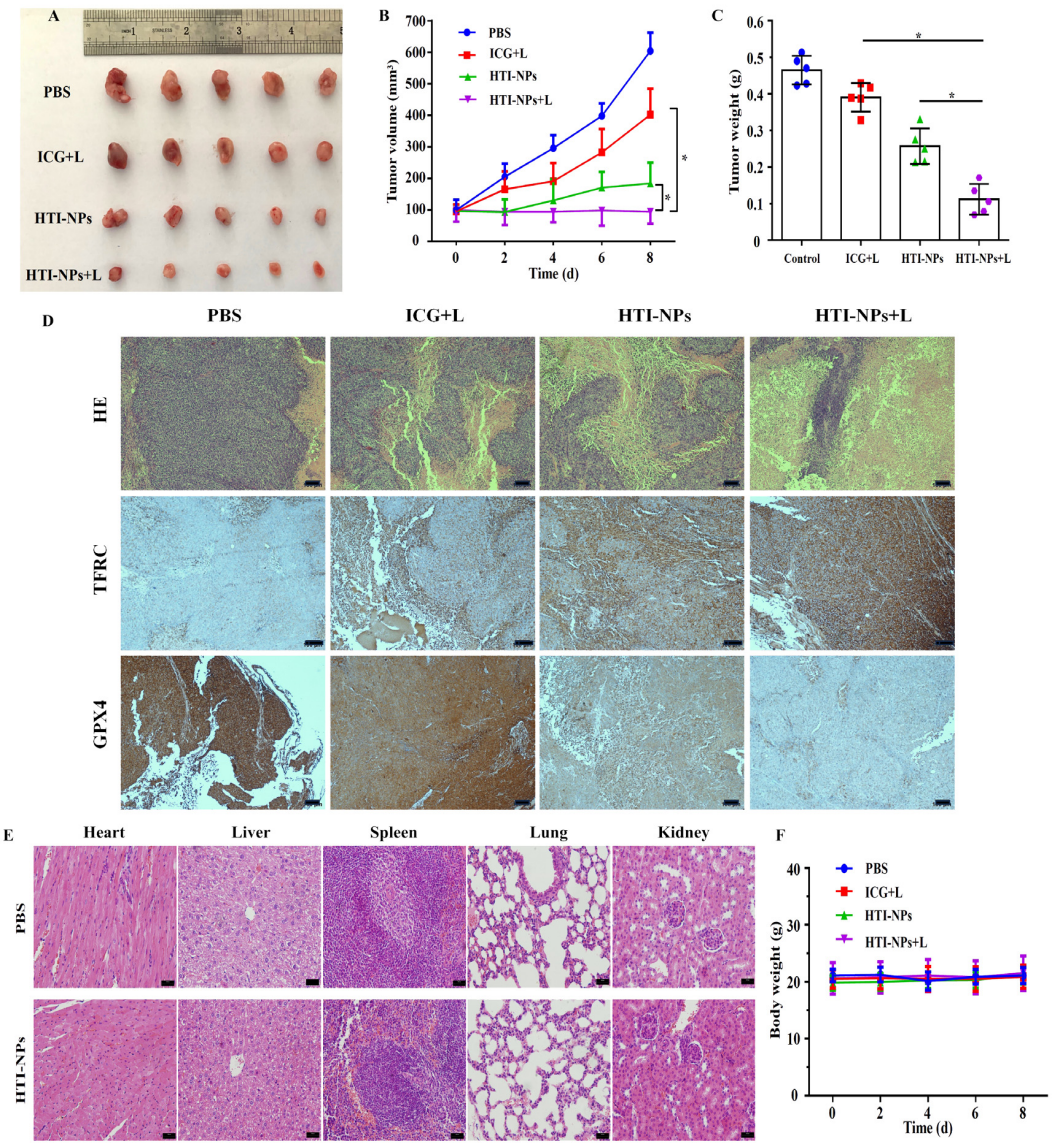
**(A)** Photograph of tumors on day 8 in different groups at the end of treatments. **(B)** Tumor volume and **(C)** tumor weight of mice in different groups over 8 d **(D)** H&E staining and immunohistochemistry (TFRC and GPX4) images of tumor in different groups at the end of treatments. **(E)** H&E staining images of major organs collected from the PBS injected mice and HTI-NPs injected mice. **(F)** The body weight of mice in different groups over 8 d. * P < 0.05.

As seen in **Figure 9A**, the tumor volumes were similar in each group at the beginning of the treatment. After 4 rounds of treatment, the tumor sizes quickly grew in the PBS groups, indicating steady growth. In contrast, the growth rates of the tumor were slower in mice treated with ICG + Laser or HTI-NPs, which could be attributed to the laser-induced PDT or nanoparticles causing ferroptosis-mediated death. The tumor sizes decreased further in the HTI-NPs+ Laser groups, which showed excellent combinatorial inhibition of tumor growth (**Figures 9B, C**). The H&E-stained tumor sections showed marked nuclear fragmentation and reduced nuclear size in the HTI-NPs + Laser treatment group compared to the ICG + Laser or HTI-NPs groups (**Figure 9D**). In addition, immunohistochemical staining results indicated that compared to other treatment groups, HTI-NPs significantly up-regulated TFRC expression and suppressed GPX4 expression in MCF-7/ADR cells after laser irradiation (**Figure 9D**).

### 3.7 Biological toxicity assessment

As shown in **Figure 9E**, the nanoparticles have a satisfactory biocompatibility, leading to no changes of body weight and few adverse effects during the 8day treatment. Additionally, we performed a histological investigation on mice to look into any potential biological harm. When compared to the PBS group, the H&E staining images of major organs from the HTI-NPs treated group showed no obvious damage or inflammation, indicating that there was no clear adverse effect on the treated mice *in vivo* as a result of HTI-NPs treatment (**Figure 9F**).

## 4 Discussion

Dysregulation of apoptosis is an important mechanism of intrinsic and acquired resistance in tumors (29). Ferroptosis can bypass apoptosis-related pathways and effectively kill drug-resistant tumor cells, making it a promising approach to overcome resistant tumors, including breast cancer (7, 30). Nanomaterials have advantages such as targeting ability and improved therapeutic index, playing a significant role in tumor treatment (31–34). By constructing iron-induced cell death nanomaterials, various types of tumors can be effectively eradicated (35–37). For instance, Jiang et al. developed photosynthetic oxygen-generating nanocapsules (PMCs) that induce lipid peroxidation, iron ion release, and downregulation of GPX4 under the action of NIR-II and X-rays, thereby eliminating radiation-resistant melanoma through ferroptosis (29). Yao et al. loaded simvastatin into magnetic nanoparticles coated with amphiphilic polymer Fe3O4@PCBMA to prolong their circulation in the blood and enhance tumor targeting. By downregulating GPX4 expression in tumor tissue and releasing a large amount of iron ions, ferroptosis was induced, resulting in the killing of triple-negative breast cancer (38). In this study, three well-recognized biocompatible materials, HSA, TOS, and ICG, were used to construct a novel ferroptosis-regulating and ROS-inducing nanomaterial called HTI-NPs. The therapeutic effect of HTI-NPs on drug-resistant breast cancer (MCF-7/ADR) was evaluated for the first time. The results demonstrated that HTI-NPs could effectively kill drug-resistant breast cancer cells through inducing ferroptosis. This research provides new insights and options for the treatment of drug-resistant breast cancer and even other resistant tumors.

Vitamin E, as a common fat-soluble antioxidant, can protect normal cell mem-branes (such as alveolar cells and red blood cells) from oxidative damage induced by free radicals (39). Furthermore, the esterified form of vitamin E (such as TOS) exhibits more stable antioxidant activity compared to the free form of vitamin E. In tumor cells, TOS can induce excessive ROS and exert anti-tumor activity by disrupting mitochondrial function (9, 40). These findings suggest that TOS can selectively induce oxidative damage in tumor cells while protecting normal cells from excessive ROS-induced damage. Studies have shown that vitamin E can regulate TFRC, affecting the content of iron ions in liver cells (13) and lipid peroxidation induced by ROS can increase the content of Fe^2+^ in the unstable intracellular iron pool (41). Therefore, it is speculated that TOS may have the potential to regulate iron ions and induce ferroptosis in tumor cells. Experimental results demonstrate that HTI-NPs can increase the expression of TFRC in MCF-7/ADR cells, induce ROS production in cells, and selectively kill drug-resistant breast cancer cells. This ferroptosis-related anticancer activity is closely associated with TOS in HTI-NPs. GPX4 is the only GPX subtype that protects biological membranes from oxidative damage and can convert toxic lipid peroxides (L-OOH) into non-toxic lipid alcohols (L-OH) using GSH. It is considered one of the markers of ferroptosis (5, 42, 43). The downregulation of GPX4 expression in MCF-7/ADR cells by HTI-NPs further confirms its role in inducing ferroptosis. Studies have found that excessive ROS can inhibit GPX4 expression by depleting GSH (44, 45) suggesting that the induction of excessive ROS by TOS in HTI-NPs is a major factor in downregulating GPX4.

Nanomaterials based on TOS have self-assembly and tumor passive targeting characteristics, which can enhance the therapeutic efficacy of TOS itself and other lipophilic drugs. To further enhance the ferroptosis-inducing properties of the nano-materials, the hydrophobic interactions between HSA, TOS, and ICG were utilized to introduce ICG onto the surface of TOS-based nanomaterials (referred to as HTI-NPs) (46). ICG is a clinically used diagnostic and therapeutic agent that, upon excitation by near-infrared light, not only exhibits fluorescence imaging capabilities but also induces cell toxicity through photodynamic and photothermal effects (47). The photodynamic effect mediated by ICG can generate various ROS, including H_2_O_2_ and singlet oxygen, through type I/II reactions and is considered a potential inducer of ferroptosis (48, 49). The combination of the fenton reaction mediated by H_2_O_2_ and Fe^2+^, the photodynamic effect of ICG, and the intrinsic activity of TOS leads to an explosive generation of ROS in HTI-NPs under near-infrared light stimulation. This process induces lipid peroxidation on one hand and enhances cell ferroptosis by depleting GSH and inhibiting GPX4 activity on the other hand. Experimental results demonstrate that HTI-NPs, upon light stimulation, further increase the intracellular ROS content and downregulate GPX4 expression in MCF-7/ADR cells, enhancing the proliferation inhibition and cytotoxicity against MCF-7/ADR cells. This suggests that the combination of ICG-mediated PDT and TOS synergistically promotes ferroptosis in drug-resistant breast cancer cells. Some researchers believe that the photothermal effect mediated by ICG is stronger than the photodynamic effect and can cause tumor cell death by generating high temperatures (50, 51). Liu et al. proposed that the aggregated form of ICG prevents its binding to surrounding oxygen molecules, resulting in the conversion of absorbed photon energy into heat energy (52). In the synthesized nanomaterial HTI-NPs, ICG is dispersed on the surface of the nanoparticles through HSA, allowing sufficient interaction between ICG and oxygen molecules in the environment. This facilitates the transfer of photon energy to oxygen molecules, leading to ROS generation primarily mediated by the photodynamic effect. Increasing research indicates that PDT and photothermal therapy have a synergistic anti-tumor effect (53–55). The research team has previously confirmed that HTI-NPs have certain photothermal effects and cytotoxicity against breast cancer. It is believed that an appropriate thermal effect can enhance cellular uptake of nanodrugs or accelerate the release of drugs from nanocarriers inside cells (17, 56). Therefore, in HTI-NPs, ICG primarily mediates photodynamic therapy and is supplemented by photothermal effects. On one hand, the photothermal effect can increase the uptake of HTI-NPs by tumor cells, and on the other hand, it synergistically enhances the photodynamic effect to induce tumor cell death.

As excellent carriers, nanoscale materials can passively target and accumulate in tumor tissues through the enhanced EPR effect, thereby increasing drug efficacy and reducing systemic toxic side effects. Fluorescence imaging demonstrates that intravenous injection of HTI-NPs leads to selective accumulation in tumor tissues. *In vivo* tumor efficacy results show that the HTI-NPs + L group significantly inhibits the growth of drug-resistant breast cancer tumors without toxic side effects on other vital organs. These effects can be attributed to several factors: the nanoscale size of HTI-NPs (275nm), the high biocompatibility of the constituent components, and the synergistic induction of ferroptosis through ICG-mediated photodynamic therapy combined with the activity of TOS.

HTI-NPs, as a novel nanomaterial for inducing ferroptosis, can also enhance the therapeutic efficacy against drug-resistant breast cancer by co-loading with other anti-tumor drugs. The hydrophobic interaction between HSA in HTI-NPs and paclitaxel enables their combination, while the stable ion pair formation between TOS and doxorubicin provides favorable conditions for the co-administration of HTI-NPs with existing clinical breast cancer chemotherapeutic drugs (17, 57). Other potential natural anti-breast cancer drugs, such as furospinulosin 1 (inducing apoptosis) (58), ROO1 (inducing pyroptosis) (59) and DMOCPTL (inhibiting GPX4, inducing ferroptosis) (60), can be loaded into the hydrophobic core region of HTI-NPs to achieve synergistic effects of apoptosis-ferroptosis, pyroptosis-ferroptosis, and dual ferroptosis against drug-resistant breast cancer. However, further research is needed to investigate the specific combination effects, *in vivo* pharmacokinetic characteristics, and toxicity profiles of these combinations.

## 5 Conclusions

In this study, we synthesized a biocompatible nanomaterial called HTI-NPs, which induces ferroptosis, and investigated its therapeutic effect on drug-resistant breast cancer for the first time. HTI-NPs can target tumor tissues and be taken up by cells. The regulation of ferroptosis by TOS, combined with the explosive growth of ROS induced by ICG-mediated photodynamic therapy and the intrinsic activity of TOS, synergistically induces ferroptosis in drug-resistant breast cancer, significantly inhibiting tumor growth without toxic side effects on other vital organs. This demonstrates the safe and effective application advantages of HTI-NPs. Furthermore, HTI-NPs also provide an ideal carrier platform for the combination therapy of drug-resistant breast cancer.

## Data Availability

The original contributions presented in the study are included in the article/supplementary material. Further inquiries can be directed to the corresponding author.
